# Using weighted power mean for equivalent square estimation

**DOI:** 10.1002/acm2.12201

**Published:** 2017-10-31

**Authors:** Sumin Zhou, Qiuwen Wu, Xiaobo Li, Rongtao Ma, Dandan Zheng, Shuo Wang, Mutian Zhang, Sicong Li, Yu Lei, Qiyong Fan, Megan Hyun, Tyler Diener, Charles Enke

**Affiliations:** ^1^ Department of Radiation Oncology University of Nebraska Medical Center Omaha NE USA; ^2^ Department of Radiation Oncology Duke University Health System Durham NC USA; ^3^ Department of Radiation Oncology The Affiliated Union Hospital Fujian Medical University Fuzhou China

**Keywords:** Akaike Information Criterion, equivalent square, linac output factors, weighted power mean

## Abstract

**Purpose:**

Equivalent Square (ES) enables the calculation of many radiation quantities for rectangular treatment fields, based only on measurements from square fields. While it is widely applied in radiotherapy, its accuracy, especially for extremely elongated fields, still leaves room for improvement. In this study, we introduce a novel explicit ES formula based on Weighted Power Mean (WPM) function and compare its performance with the Sterling formula and Vadash/Bjärngard's formula.

**Methods:**

The proposed WPM formula is $ES^{WPM}\left(a,b\right)={\left(w\;a^{\alpha }+\left(1-w\right)\;b^{\alpha }\right)}^{1/\alpha }$ for a rectangular photon field with sides a and b. The formula performance was evaluated by three methods: standard deviation of model fitting residual error, maximum relative model prediction error, and model's Akaike Information Criterion (AIC). Testing datasets included the ES table from British Journal of Radiology (BJR), photon output factors (*S*_*cp*_) from the Varian TrueBeam Representative Beam Data (Med Phys. 2012;39:6981–7018), and published *S*_*cp*_ data for Varian TrueBeam Edge (*J Appl Clin Med Phys*. 2015;16:125‐148).

**Results:**

For the BJR dataset, the best‐fit parameter value α = −1.25 achieved a 20% reduction in standard deviation in ES estimation residual error compared with the two established formulae. For the two Varian datasets, employing WPM reduced the maximum relative error from 3.5% (Sterling) or 2% (Vadash/Bjärngard) to 0.7% for open field sizes ranging from 3 cm to 40 cm, and the reduction was even more prominent for 1 cm field sizes on Edge (*J Appl Clin Med Phys*. 2015;**16**:125–148). The AIC value of the WPM formula was consistently lower than its counterparts from the traditional formulae on photon output factors, most prominent on very elongated small fields.

**Conclusion:**

The WPM formula outperformed the traditional formulae on three testing datasets. With increasing utilization of very elongated, small rectangular fields in modern radiotherapy, improved photon output factor estimation is expected by adopting the WPM formula in treatment planning and secondary MU check.

## 1 INTRODUCTION

Equivalent Square (*ES*) is a widely used and important concept in photon external beam radiation dose calculation. ES postulates that, for an arbitrary rectangular field, there exists an equivalent square field sharing certain dosimetric characteristics. That concept provides us with a pathway for estimating a rectangular field's properties (e.g., central axis percentage depth dose, scatter factor) from measurements performed on square fields.

The crucial step in the success of this approach is to identify the optimal formula that will predict the correct equivalent square. For a rectangular field with width *a* and length *b*, Sterling's formula^1^

(1) $$ES^{Sterling}\left(a,b\right)=\frac{2\;a\;b}{a+b},$$

was historically the first widely used, explicit *ES* formula for such a purpose. It remains the primary choice in current medical physics practice. Originally proposed in 1964 for studying the rectangular radiation field's central axis percentage depth dose that is generated by X‐ray units and ^60^Co machines, Sterling's formula has enjoyed success from that point to this, and now is almost a synonym for *ES* because of its simple mathematical structure and good prediction power for conventionally shaped and sized fields in many applications.

The next major milestone in explicit *ES* formula history occurred about thirty years after the introduction of the Sterling's formula. To include the collimator exchange effect observed in their study of linear accelerator (linac) head‐scatter factors, Vadash and Bjärngard (VB) presented their modified version of Sterling's *ES,* 2, 3 specifically

(2) $$ES^{VB}\left(a,b\right)=\frac{\left(A+1\right)\;a\;b}{A\;a+b},$$

with an adjustable parameter *A* > 0.

Compared with Sterling's formula, this new form explicitly accounts for the collimator exchange effect.^4^ It can also reduce the maximum discrepancy in linac rectangular field head‐scatter factor prediction, down to about 1% for the resulting clinically relevant field sizes.^2^

The radiation oncology field has recently witnessed remarkable developments in the technology of radiotherapy delivery. Flattening‐filter‐free photon mode has become commonplace; very small and elongated photon fields are frequently used in both the IMRT and VMAT delivery processes. When dealing with these extreme situations, use of the previous *ES* formulae may lead to worrisome discrepancies. Thus, current radiation delivery modalities warrant an update to the explicit *ES* formula.

Therefore, the aim of this study, using two well‐known datasets, was to propose a revised formula and demonstrate that it offers an improvement over the two most popular, conventional formulae.

## 2 METHODS

Herein, a Weighted Power Mean (*WPM*)^5^, ^6^ based ES formula was introduced, with the aim of achieving better accuracy than that obtained by the Sterling's and VB's formulae:

(3) $$ES^{WPM}\left(a,b\right)={\left(w\;a^{\alpha }+\left(1-w\right)\;b^{\alpha }\right)}^{1/\alpha }$$

with two adjustable parameters: power index α and weighting factor $w\in \left(0,1\right)$.

It is worth mentioning that our formula [see Eq. (3)] can be reduced to Sterling's formula (α ≡ −1 and $w\equiv \frac{1}{2}$) [see Eq. (1)] or VB's formula (α ≡ −1 and $w=\frac{1}{1+A}$) [See Eq. (2)]. We included these two cases in our parameter selection, therefore the proposed formula is guaranteed to fit any dataset no worse than the two well‐known formulae would.

By definition, all *ES* formulae should give *a* when a rectangular field degenerates to a square field (i.e. b→a). Our *WPM*‐based *ES* certainly satisfies this requirement. With any positive field size *a*, no matter what the values of α and *w* are:

(4) $$ES^{WPM}\left(a,a\right)={\left(w\;a^{\alpha }+\left(1-w\right)\;a^{\alpha }\right)}^{1/\alpha }=a.$$

We tested this new *WPM‐*based *ES* formula on two publicly available datasets: (a) tables of equivalent square fields for central axis dose calculations in the *British Journal of Radiology* (*BJR*), supplement 25^7^; and (b) in‐water output factor ratios (*S*_*cp*_) from the Varian TrueBeam Representative Beam Data for Eclipse (Varian Medical Systems, Palo Alto, CA, USA).^8^ The latter provided the *S*_*cp*_ measurements for linac jaw‐defined rectangular and square fields with field size between 3 cm and 40 cm in an *SAD* setup at *SSD* = 95 cm, with the ion chamber placed in the center of the photon field at 5 cm in‐water depth.

We considered two methods for parameter fitting of the proposed formula:

1. Performing a nonlinear least‐squares fitting to determine parameter values (i.e., *w* and α) based on known $ES\left(a,b\right)$ values for rectangular fields with different size combinations $\left\{a,b\right\}$:

(5) $$\left\{w,\alpha \right\}=\underset{\left\{w\geq 0,\alpha \right\}}{\arg\min}\left(\sum_{\left\{a,b\right\}}{\left(ES\left(a,b\right)-{\left(wa^{\alpha }+\left(1-w\right)b^{\alpha }\right)}^{1/\alpha }\right)}^{2}\right)$$

**Figure 1:**
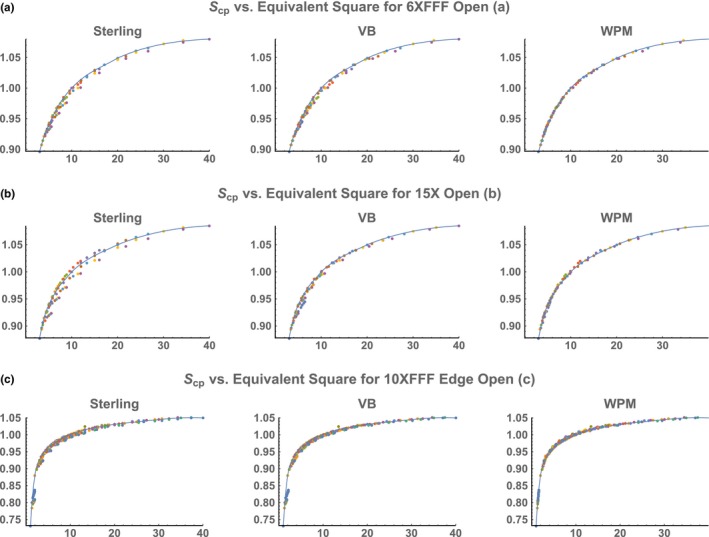
*S*_*cp*_ as a function of estimated *ES* for various photon energies [(a) 6X FFF, (b) 15X, and (c) 10X FFF Edge]. Open‐field *S*_*cp*_ values from the Varian representative dataset and the published Edge dataset were plotted as a function of *ES* predicted by three models. The solid lines in the plots are cubic splines with construction based only on square‐field measurements.

1. Given a radiation property *R* and its measured values *R(a,b)*, from a radiation generating device with rectangular/square field size combinations $\left\{a,b\right\}$, *w* and α are determined through the following numerical procedure: since ES≡a when *a* = *b* [See Eq. (4)], in the Rvs.ES plot, all square field measurement points will be fixed points when we adjust the parameters *w* and α in the *ES* formula [See Eq. (3)]. As a result, we can set the constructed cubic‐spline (*CS*)^9^ based on the square field measurement points only (See solid lines in Fig. 1) as our target curve, and select the two parameters α and *w* values as those that minimize the sum of the squares of the difference between $R\left(a,b\right)$ and the predicted value $CS\left(ES\left(a,b\right)\right)$ for all rectangular field measurements.

With the *BJR* supplement 25 dataset, where the target $ES\left(a,b\right)$ value was given (as in Scenario 1 above), the standard deviation of the residual error after nonlinear least‐square fitting was used to judge the quality of the *ES* formula.

For S_cp_ in the Varian dataset (method 2 above) and the published measurement data from a Varian TrueBeam Edge unit,^10^ the quality of an ES formula was visualized in the $S_{\mathrm{cp}}\;\mathrm{vs}.\;\mathrm{ES}$ plot. The reason for this was that if an ES formula correctly identified the underlying relationship between a rectangular field and its corresponding ES measured in S_cp_, then all data points, no matter whether they were from square field measurements or rectangular field measurements, should fall onto the same curve in a plot of $S_{\mathrm{cp}}\;\mathrm{vs}.\;\mathrm{ES}$. Clinically, the maximum absolute value in relative error between the predicted and the measured S_cp_ value for an ES formula is most relevant to radiotherapy delivery (specifically MU calculation) and, therefore, can be used as a criterion for clinical model comparison.

The Akaike Information Criterion (*AIC*)^11^ is a statistical tool for comparing different model performances on the same dataset wherein models are allowed to use different numbers of free parameters. For the *ES* formulae presented here, there are zero, one, and two free parameters in the Sterling, VB, and our *WPM* based formula respectively. The relative score of a model's performance can be expressed as:

(6) AIC=2k−2ln(L),

where *k* is the number of free parameters in the formula, and *L* is the maximum value of the likelihood function for the formula. Under the assumption that the residuals are distributed according to independent identical normal distributions (with zero mean), we have

(7) $$AIC=2k+n\;\text{ln}\left(\frac{RSS}{n}\right)+\mathrm{constant}.$$

Here, *n* is the number of data points, $RSS=\sum_{i=1}^{n}{\left(y_{i}-f\left(x_{i}|\theta \right)\right)}^{2}$ is the residual sum of squares for formula f with the optimal parameter set θ, and the constant term is model‐independent for a given dataset. We applied *AIC* as an objective comparison between our formula and the two established formulae. Please note that for the same dataset, only the relative value of *AIC* is meaningful; therefore, we will set the constant term to zero for the rest of this paper. For model comparison, the lower the *AIC* value, the better the model's performance.

## 3 RESULTS

When we applied the *WPM*‐based formula to the BJR *ES* table, due to the intrinsic symmetry between the two field sides of every rectangular field in the dataset, β must be $\frac{1}{2}$. Next, we performed least squares fitting to obtain the optimal α value:

(8) $$\alpha =\underset{\alpha }{\arg\min}\left(\sum_{\left\{a,b\right\}}{\left(ES\left(a,b\right)-{\left(\frac{a^{\alpha }+b^{\alpha }}{2}\right)}^{1/\alpha }\right)}^{2}\right)$$

where $ES\left(a,b\right)$ is the known ES for a rectangular field with sizes a and b.

For the *BJR* dataset, the fitting procedure above led to α=−1.25, and the standard deviation of residual error was reduced by 20% compared with using α = −1 (i.e., the Sterling‐type *ES* formulae). The 99% confidence interval of α was $CI_{99\%}=\left[-1.34,-1.16\right]$. The optimal value for α was unlikely to be −1 for this dataset. Therefore, using the *BJR* dataset to support the use of Sterling's formula was based more upon clinical practicality rather than statistical analysis.

The best fitting values of α and *w* for open field *S*_*cp*_ in the Varian dataset for Eclipse are listed in Table 1 for some representative photon energies. Again, none of them selected α = −1.

**Table 1:** The best fitting values of parameters α and *w* for open field *S*_*cp*_ of selected photon energies from the Varian dataset

Fitting parameter	Beam energy/mode			
	6X	15X	6X FFF	10X FFF
α	−1.17	−1.40	−1.32	−1.49
w	0.56	0.59	0.53	0.56

All open‐field values of *S*_*cp*_ from the Varian dataset were plotted against the predicted *ES* values from the three explicit *ES* formulae for four different photon energies (See Fig. 1). We can see that our *WPM* formula did a better job minimizing the spread of data points around the measured square‐field curve, indicating better modeling performance.

**Figure 2:**
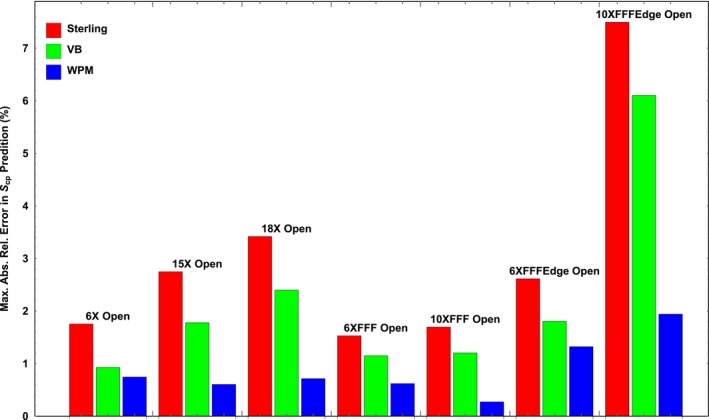
Maximum absolute value in relative error in open‐field *S*_*cp*_ prediction from the three models for various photon energies are listed in the Varian dataset and the reference.^10^

The largest magnitude relative errors for open fields at all photon energies from the Varian dataset were graphed in Fig. 2 for all three *ES* formulae. We observed the same order of performance for all photon energies: *WPM* performed the best, followed by VB's, and then Sterling's.

**Figure 3:**
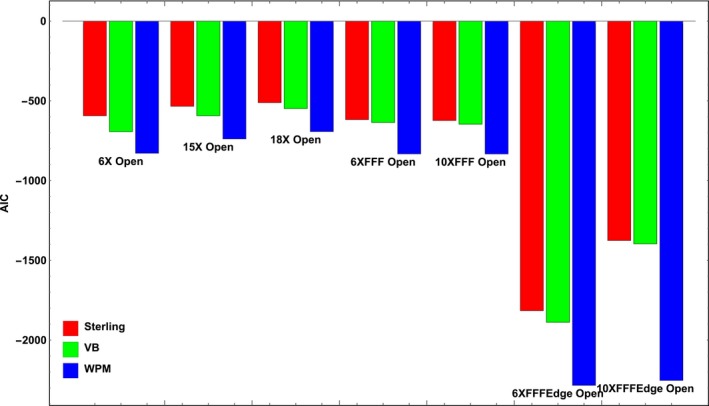
Relative *AIC* values of the three *ES* formulae are computed based on some open field photon *S*_*cp*_ measurements from the Varian dataset and the reference.^10^ In all cases, the *WPM* model performed the best, and Sterling the worst.

The more rigorous comparison was performed based on the Akaike theory. The *AIC* value of the *WPM* formula was consistently lower than its counterparts from the traditional methods when we performed model fitting on the photon open field *S*_*cp*_ tables in the Varian dataset (See Fig. 3). The lowest AIC values among the three formulae indicated that the newly proposed *WPM*‐based *ES* formula outperformed both the Sterling and VB formulae, even after we took into account the number of adjustable model parameters.

The worst relative data fitting errors for all three formulae occurred at data entries where photon fields were very elongated, narrow fields. The relative model fitting error for the fields with a shorter side at 1cm is tabulated in Table 2. We interpolated Varian 10X FFF Edge *S*_*cp*_ values of nonsquare shaped fields based only on the square field measurements and *ES* predicted by the three formulae. The improvements of the *WPM* formula over the other two traditional formulae were obvious for these hard‐to‐fit field cases of large aspect ratios and very small short side lengths. From Table 2, we can see that the VB's formula has lower maximum relative error than the Sterling's formula while the *WPM* consistently outperformed the other two traditional *ES* formulae when it was applied to elongated small fields.

**Table 2:** Model prediction relative error for the three ES formulae when they are applied to *S*_*cp*_ collected from 10X FFF fields with a short side 1 cm in length on a Varian TrueBeam Edge unit.^10^

10X FFF edge data	Relative prediction error in *S*_cp_ (%)					
Field long side (cm)	Sterling		VB		WPM	
	X Jaw size = 1 cm	Y Jaw size = 1 cm	X Jaw size = 1 cm	Y Jaw size = 1 cm	X Jaw size = 1 cm	Y Jaw size = 1 cm
1	0.0	0.0	0.0	0.0	0.0	0.0
2	1.7	0.3	0.9	0.5	0.4	0.5
3	3.5	1.2	2.3	2.3	0.1	0.1
4	4.5	2.2	3.2	3.6	0.1	0.5
5	5.7	3.0	4.3	4.4	0.4	0.7
6	6.1	3.3	4.6	4.8	0.3	0.7
7	6.4	3.7	4.9	5.2	0.3	0.7
8	6.8	4.0	5.2	5.5	0.4	0.7
10	7.1	4.1	5.5	5.7	0.2	0.5
12	7.6	4.6	5.9	6.2	0.4	0.7
15	7.6	4.4	6.0	6.0	0.2	0.3
20	8.0	4.5	6.3	6.1	0.3	0.2
25	8.1	4.8	6.4	6.5	0.2	0.3
30	8.1	5.1	6.4	6.7	0.1	0.4
35	8.3	5.1	6.6	6.7	0.2	0.3
40	8.3	4.6	6.5	6.3	0.1	0.1

When other factors are fixed, the relative error in dose calculation for a photon field equates to the relative error in the employed output factor. Therefore, we expect our new *ES* formula will improve the accuracy of dose calculation, particularly when the photon field has an elongated small rectangular shape.

## 4 DISCUSSION

Both the Sterling formula and the Vadash and Bjärngard formula have been used clinically for many decades. They are both mathematically simple and, more importantly, provide good approximations of *ES* for rectangular clinical treatment fields when the field shape is not too far away from a square. Rapid technical advances in radiotherapy, especially the use of elongated small treatment fields, have advanced radiotherapy in many ways. This provided us with the motivation to propose a new *ES* estimation formula for the new generation of radiotherapy, similar to the case that the collimator exchange effect motivated the introduction of the VB formula thirty years ago. The proposal we presented here is a natural generalization of the two well‐established formulae. Comparing with the generic classical formulae, the new formula contains additional variables which could be optimized for best prediction accuracy based on LINAC type and beam energy. However, tests using the AIC criteria presented in this work unequivocally established the superiority of the new formula, especially in clinical scenarios enabled by modern technological revolutions. Introducing a mathematically more complex *WPM* formula with an additional fitting parameter (i.e., α) comes with a relatively modest price and is justifiably offset by the gain in reducing the systematical error in *S*_*cp*_ prediction for small elongated rectangular fields such as those presented in Table 2. The success of our proposed formula on the two widely used datasets revealed its compelling clinical potential to alleviate the number of required commissioning measurements, while maintaining the quality of beam data for TPS modeling. It could also improve the accuracy of secondary MU check, which currently often suffers from unacceptable accuracy in calculating IMRT and VMAT plans. Further validation may be necessary for linear accelerators from other vendors.

## 5 CONCLUSIONS

A novel *WPM* formula has been proposed for *ES* estimation, which outperformed Sterling's explicit *ES* formula and its variant proposed by Vadash and Bjärngard on two well‐known public datasets. Both the weighting factor and the power index in the *WPM* formula can be determined through simultaneous optimization to achieve better accuracy. The improvement of the *WPM* over the Sterling‐type explicit *ES* formulae is particularly obvious for very elongated small rectangular fields that have been used with increasing frequency in IMRT and VMAT delivery. Improved dose calculation accuracy is expected when the *WPM* formula is adopted into treatment planning and secondary MU check systems.

## ACKNOWLEDGMENTS

The authors would like to thank Varian Medical Systems for research grant support.

## CONFLICTS OF INTEREST

This project was funded by Varian Medical Systems.
